# Moderation effects of health behaviors on stress and suicidal ideation in adolescents: a cross-sectional survey during COVID-19

**DOI:** 10.1038/s41598-023-48972-y

**Published:** 2023-12-04

**Authors:** Teresa O’Rourke, Elke Humer, Paul L. Plener, Christoph Pieh, Thomas Probst

**Affiliations:** 1 Department of Psychosomatic Medicine and Psychotherapy, Danube University Krems, Dr.-Karl-Dorrek-Str. 30, 3500 Krems, Austria; 2 Faculty of Psychotherapy Science, Sigmund Freud University Vienna, 1020 Vienna, Austria; 3 Department of Child and Adolescence Psychiatry, Medical University of Vienna, 1090 Vienna, Austria; 4 Department of Child and Adolescent Psychiatry and Psychotherapy, University of Ulm, 89075 Ulm, Germany; 5 Division of Psychotherapy, Department of Psychology, University of Salzburg, 5020 Salzburg, Austria

**Keywords:** Risk factors, Psychology, Human behaviour

## Abstract

This cross-sectional online survey study investigated whether certain health behaviors moderated the relationship between perceived stress and suicidal ideation in Austrian adolescents during the COVID-19 pandemic. A total of 1505 14–20-year-old (median age = 16) high school students (77.9% female) filled out an online survey from September to November 2021. Perceived stress was measured with the PSS10, suicidal ideation with item 9 of the PHQ-9. The following health behaviors were assessed: Physical activity (days/week), phone use (hours/day), problematic drinking behavior (CAGE). All three health behaviors significantly moderated the relationship between perceived stress and suicidal ideation (all *p* < .05), but effects were small. The moderation analyses revealed that higher physical activity and less time spent on the phone were associated with less suicidal ideation at higher stress levels. Showing signs of problematic drinking behavior was associated with higher suicidal ideation at higher stress levels. In conclusion, these results suggest that some health behaviors may be able to act as a buffer between perceived stress and suicidal ideation. However, more research is needed to confirm these potentially buffering effects.

## Introduction

The COVID-19 pandemic and the associated restrictions implemented to prevent an uncontrolled dissemination of the disease have disrupted the daily lives of people all over the world. The psychosocial consequences of these pandemic-related restrictions are becoming increasingly evident in the general population^1^, but particularly in children and adolescents^2^. Adolescence is characterized by crucial social and cognitive development^3^, which has been directly impacted by measures such as school closures and subsequent home-schooling. Schools constitute an important environment for adolescent development by providing context for peer-interactions essential for developing social identity and autonomy^4^. By having to adapt to distance learning, many adolescents not only experienced a loss of social contact with their peer-group, but both students and their parents also had to change daily routines, which resulted in many parents experiencing high levels of stress during the pandemic^5^. As consequences of these restrictions, many adolescents reported school-related stress, such as difficulties completing school projects and pressure to perform, increased interpersonal problems with family and friends, and worries about the future^6^. A study conducted in 2015 emphasized the multidimensionality of stressors in university students by identifying academic performance, pressure to succeed, and post-graduation plans as students most mentioned concerns even in non-pandemic times^7^. It is highly likely, that the pandemic-related restrictions have exacerbated these concerns for many students. After one semester of home-schooling in Austria, over 50% of 2884 surveyed high school students reported experiencing moderate stress levels, and one third reported experiencing high stress-levels^8^. Such an accumulation of stressors over time can exacerbate mental health symptoms^9^, which is especially worrisome in this age group, as adolescence already constitutes a vulnerable time for the development of mental health disorders^10^. For many individuals, adolescence and the transition to early adulthood constitutes a highly stressful time, with a meta-analysis from 2020 revealing an alarmingly high prevalence of stress, anxiety, and depression in university students^11^. The loss of social contacts through social distancing also brought with it a loss of a crucial coping strategy, namely social support, which has been linked to higher quality of life and well-being, as well as lower perceived stress, depressive symptoms, anxiety, and insomnia during COVID-19^12^. A lack of adaptive coping-strategies, on the other hand, has been associated with experiencing chronic stress^13^ and may constitute a further risk factor for the development of mental disorders. Further risk factors for mental health problems can be adverse health behaviors, which have reportedly increased following the onset of the pandemic due to disruptions in daily life activities and the subsequent change in lifestyle^14^. For example, a study with more than 7000 Austrian adolescents reported a significant decrease of physical activity as well as a significant increase of mobile phone use compared to pre-pandemic data^15^. This is an alarming development, as an international study conducted at the beginning of the pandemic revealed significantly worse mental health indicators in individuals exhibiting negative changes in exercise behavior following pandemic-related restriction measures, in comparison to individuals adapting to restriction measures with positive changes in exercise behavior^16^. Another alarming change in health behavior after the onset of the pandemic was the consumption of drugs. In Austrian adolescents and young adults, the most used drugs are nicotine and alcohol^17^, followed by cannabis with a lifetime prevalence of 30–40%, and ecstasy, cocaine, and amphetamines with lifetime prevalences of around 6%^18^. Pre-pandemic data from the ESPAD study reveals that while only 15% of Austrian adolescents reported to have never consumed alcohol before, this number of abstinent adolescents has increased over the last decade^19^. However, post-pandemic data from a recent general population survey reveals that around 40% of 15–34-year-old Austrians increased their alcohol consumption during the first lockdown in March 2020^15^. These changes in drinking behavior were of short duration as alcohol consumption in this age group returned to base levels after a few months, which suggests that this was a way of coping with the demands of the first lockdown for this demographic group. While alcohol consumption as a coping strategy can have short-term stress-reducing effects^20^, it leads to detrimental physical and mental health outcomes in the long run^21^. In a study conducted during COVID-19 lockdown, alcohol consumption as a coping strategy positively predicted perceived stress, symptoms of depression, anxiety, and insomnia, and negatively predicted quality of life and well-being^12^.

An alarming development during the COVID-19 pandemic in Austria was an increase in suicidal ideation in adolescents, continuing even after restrictions had been lifted. While adolescents’ suicidality did not seem to have been impacted by the onset of the COVID-19 pandemic or has seemingly even decreased during the pandemic in some countries such as Japan or New Zealand^22^, the prevalence of suicidal ideation in Austrian adolescents increased slightly from 35.3% in girls and 30.6% in boys in spring 2021, to 47.2% in girls and 33.5% in boys in autumn 2021^23^.

Perceived stress^24,25^, as well as stressful life events such as family stressors or academic stressors^26^, have been previously identified as predictors for suicidal ideation, and may have contributed to this development in some form. According to the Narrative-Crisis Model of suicide, experiencing stressful life events increases the likelihood of already vulnerable individuals of developing the Suicidal Crisis Syndrome, culminating in a sense of entrapment and little hope for the future, social withdrawal, affective disturbance, and hyperarousal^27^. The Suicidal Crisis Syndrome, in turn, has been linked to suicidal ideation as well as imminent suicidal behavior^28^. Taking this model into account, in a time of increased stress such as the COVID-19 pandemic, it is highly relevant to investigate potential protective factors for suicidal ideation. In this regard, health behaviors have been associated not only with physical health but mental health benefits as well. Physical activity, for example, may be such a protective factor for suicidal ideation, according to the findings of a meta-analysis^29^. Furthermore, multiple studies suggest a link between excessive phone use and suicidal ideation^30^ as well as self-harm^31^ in adolescents. Especially late-night phone use may be a risk factor for depression and suicidality in young people^32^. Chen et al.^33^ identified a mediating effect of interpersonal problems, a common stressor, on the relationship between excessive phone use and suicidal behaviors as well as self-harm in adolescents. In their review, Carballo et al.^26^ also identified alcohol use as a risk factor for suicidality. Both alcohol consumption as means of coping and heavy episodic drinking have been associated to increased suicidality in adolescents^34^.

In the present study, we aimed to examine whether the strength of the association between perceived stress and suicidal ideation is influenced by health behaviors. We, therefore, investigated three health behaviors (physical activity, mobile phone use, alcohol consumption) as moderators of the link between perceived stress and suicidal ideation.

The following research questions were addressed:

- RQ1 Does physical activity moderate the relationship between perceived stress and suicidal ideation?
- RQ2 Does phone usage moderate the relationship between perceived stress and suicidal ideation?
- RQ3 Does problematic drinking behavior moderate the relationship between perceived stress and suicidal ideation?

## Materials and methods

### Study design

A cross-sectional online survey was conducted from the 14th of September 2021 to the 14th of November 2021 via REDCap^35^, which is hosted at servers of the University for Continuing Education, Krems. Recruitment was conducted by school representatives, who posted a link to the online survey on various social media platforms, inviting Austrian high school students to participate. Participation in the study was voluntary. At the time of the survey, there was no lockdown in place and schools were fully open. Regular COVID-19 tests and FFP-2 masks outside of the classroom were required as protective measures in schools. To partake in the study, all participants had to give electronic informed consent by agreeing to the data protection declaration and confirm that they were at least 14 years old, as the legal age of consent in Austria is 14. This study procedure was approved by the Ethics Committee of the University for Continuing Education Krems (protocol code EK GZ 41/2018–2021) as well as a data protection officer and conducted in accordance with the Declaration of Helsinki. Further studies have been published based on this dataset^23^.

## Materials

### Perceived stress

Perceived stress was measured with the reliable and validated Perceived Stress Scale (PSS-10)^36^. The PSS-10 is a widely used self-rating questionnaire containing 10 items on a 5-point Likert scale and scores ranging from 0 to 40. Scores from 0 to 13 indicate a low stress level, scores from 14 to 26 indicate a moderate stress level, and scores from 27 to 40 indicate a high stress level. The German version has been validated for individuals aged 14 to 90^37^. The Cronbach’s α for the PSS-10 in our sample was 0.861. 

## Suicidal ideation

Suicidal ideation was assessed with the ninth item of the PHQ-9^38,39^: “Over the last two weeks, how often have you been bothered by thoughts that you would be better off dead or of hurting yourself in some way?” Possible answer options were not at all (0), several days (1), more than half the days (2), and nearly every day (3). A cut-off score of 1 is generally used to indicate suicidal ideation. The PHQ-9 has been shown to be a robust and age-independent predictor of suicide attempts and deaths^39^.

### Physical activity (Moderator 1)

To assess physical activity, participants were asked: “On how many of the last seven days have you been physically active for at least 60 min?” Possible answers ranged from 0 to 7 days.

### Phone usage (Moderator 2)

Smartphone usage was assessed by asking participants: “In a typical day, how much time do you spend–sitting or lying down–on your smartphone?” The following answers were possible: less than 1 h per day, 1–2 h per day, 3–4 h per day, 5–6 h per day, 7–8 h per day, more than 8 h per day.

### Problematic drinking behavior (Moderator 3)

Problematic drinking behavior was assessed using the validated and reliable screening questionnaire CAGE^40,41^. The CAGE consists of four yes/no questions regarding signs of alcoholism. Problematic drinking behavior is indicated when a cut-off of two or more questions have been answered with “yes”^42^.

### Statistics

To answer the three research questions, three moderation models were conducted using model 1 (one moderator) of the SPSS Macro Process. Using the Johnson-Neyman method, we tested for possible significance transition points within the observed range of the moderators. The first model tested the effect of physical activity on the relationship between perceived stress and suicidal ideation:

$$ Suicidal\;Ideation = \beta_{0} + \beta_{1} stress + \beta_{2} physical\;activity + \beta_{3} stress \times physical\;activity + \varepsilon $$

The second model tested the effect of phone use on the relationship between perceived stress and suicidal ideation:

$$ Suicidal\;Ideation = \beta_{0} + \beta_{1} stress + \beta_{2} phone\;use + \beta_{3} stress \times phone\;use + \varepsilon $$

The third model tested the effect of problematic alcohol consumption on the relationship between perceived stress and suicidal ideation:

$$ \begin{aligned} Suicidal\;Ideation = & \beta_{0} + \beta_{1} stress + \beta_{2} problematic\;alcohol\;consumption \\ & + \beta_{3} stress \times problematic\;alcohol\;consumption + \varepsilon \\ \end{aligned} $$

Gender and age were included as covariates in all 3 models. SPSS v28 was used for all analyses, all statistical tests were performed two-tailed, and the significance level was set to *p* < 0.05. To account for type-I error inflation due to multiple testing, an additional Bonferroni-adjusted significance level of *p* < 0.01666 was calculated. 

## Results

### Sample

Following the American Association for Public Opinion Research (AAPOR) reporting guidelines for nonprobability online samples^43,44^, we report that 62.31% of those who clicked on the survey link completed the entire survey, resulting in a 62.31% participation rate (ie, completion rate). In total, 1505 high school students with a mean age of 16.3 (SD = 1.4) completed the survey. 1173 (77.9%) out of these were female, 281 (18.7) male, and 51 (3.4%) reported diverse gender.

*Results for RQ1*: Does physical activity moderate the relationship between perceived stress and suicidal ideation?

**Figure 1:**
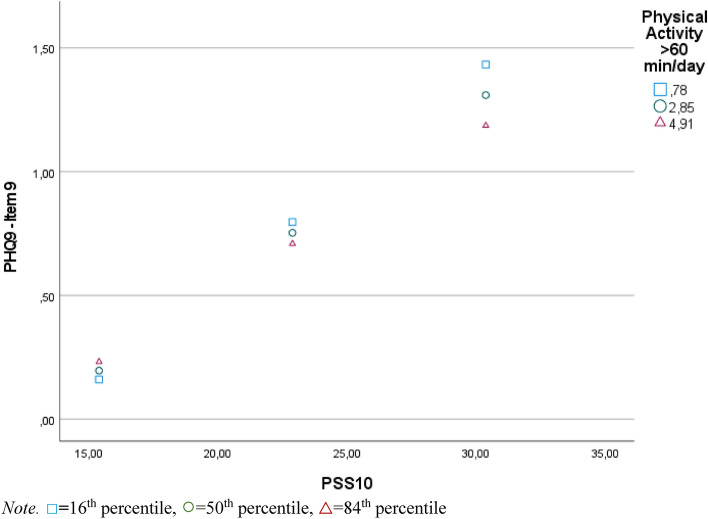
Moderation effect of physical activity on the relationship between perceived stress and suicidal ideation.

A moderation analysis was run to determine whether the interaction between perceived stress and physical activity significantly predicts suicidal ideation. The overall model was significant, F(5, 1499) = 145.57, *p* < 0.001, R = 0.572, predicting 32.68% of the variance. All predictors were significant before Bonferroni-adjusting the significance level (perceived stress = 0.089, *p* < 0.001, 95% CI [0.080, 0.099]; physical activity = 0.074, *p* = 0.021, 95% CI [0.011, 0.137]), but the *p*-value of physical activity surpassed the Bonferroni-adjusted significance level of 0.01666 However, physical activity moderated the effect between perceived stress and suicidal ideation significantly, ΔR^2^ = 0.46%, F(1, 1499) = 10.32., *p* = 0.001, 95% CI [−0.007, −0.002]. The R-Square change value indicates a small effect size. The interaction plot (Fig. 1) reveals that as stress levels increased, adolescents who were more physically active showed less suicidal ideation than less active adolescents. There were no statistical significance transition points within the observed range of the moderator found using the Johnson-Neyman method, as the effect was significant for all observed moderator values.

*Results for RQ2*: Does phone usage moderate the relationship between perceived stress and suicidal ideation?

**Figure 2:**
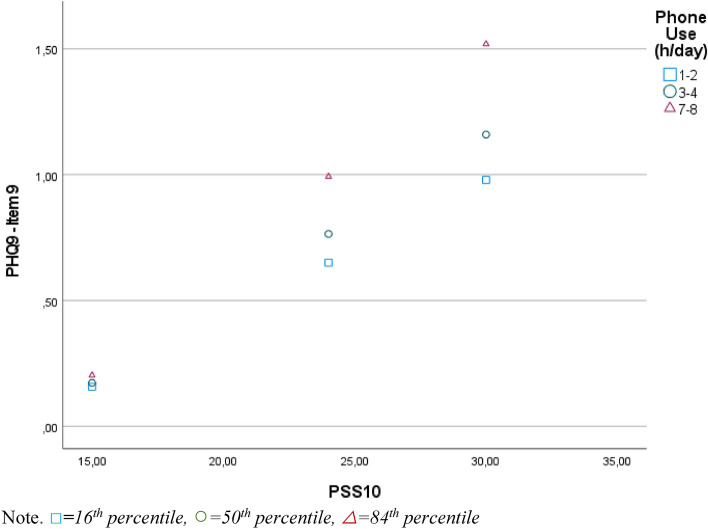
Moderation effect of phone usage on the relationship between perceived stress and suicidal ideation*.*

A moderation analysis was run to determine whether the interaction between perceived stress and phone usage significantly predicts suicidal ideation. The overall model was significant, F(5, 1499) = 155.12, *p* < 0.001, R = 0.584, predicting 34.10% of the variance. Perceived stress (estimate = 0.039, *p* < 0.001, 95% CI [0.021, 0.057]) reached statistical significance before and after the Bonferroni-correction, while phone usage (estimate = −0.123, *p* = 0.052, 95% CI [−0.247, 0.001]) was not a significant predictor for suicidal ideation alone. However, physical activity moderated the effect between perceived stress and suicidal ideation significantly, ΔR^2^ = 0.66%, F(1, 1499) = 14.91, *p* = 001, 95% CI [0.005, 0.015]. The R-Square change value indicates a small effect size. The interaction plot (Fig. 2) shows that at higher stress levels, adolescents who spent more time on their phones reported more suicidal ideation than those who spent less time on their phones. This effect becomes especially apparent at a high stress level, where adolescents who only spent 2 h per day on their phones did not meet the cut-off score for suicidal ideation (PHQ9 ≥ 1), while those who spent 3 or 5 h per day on their phone did show signs of suicidal ideation. There were no statistical significance transition points within the observed range of the moderator found using the Johnson-Neyman method, as the effect was significant for all observed moderator values.

*Results for RQ3* Does problematic drinking behavior moderate the relationship between perceived stress and suicidal ideation?

**Figure 3:**
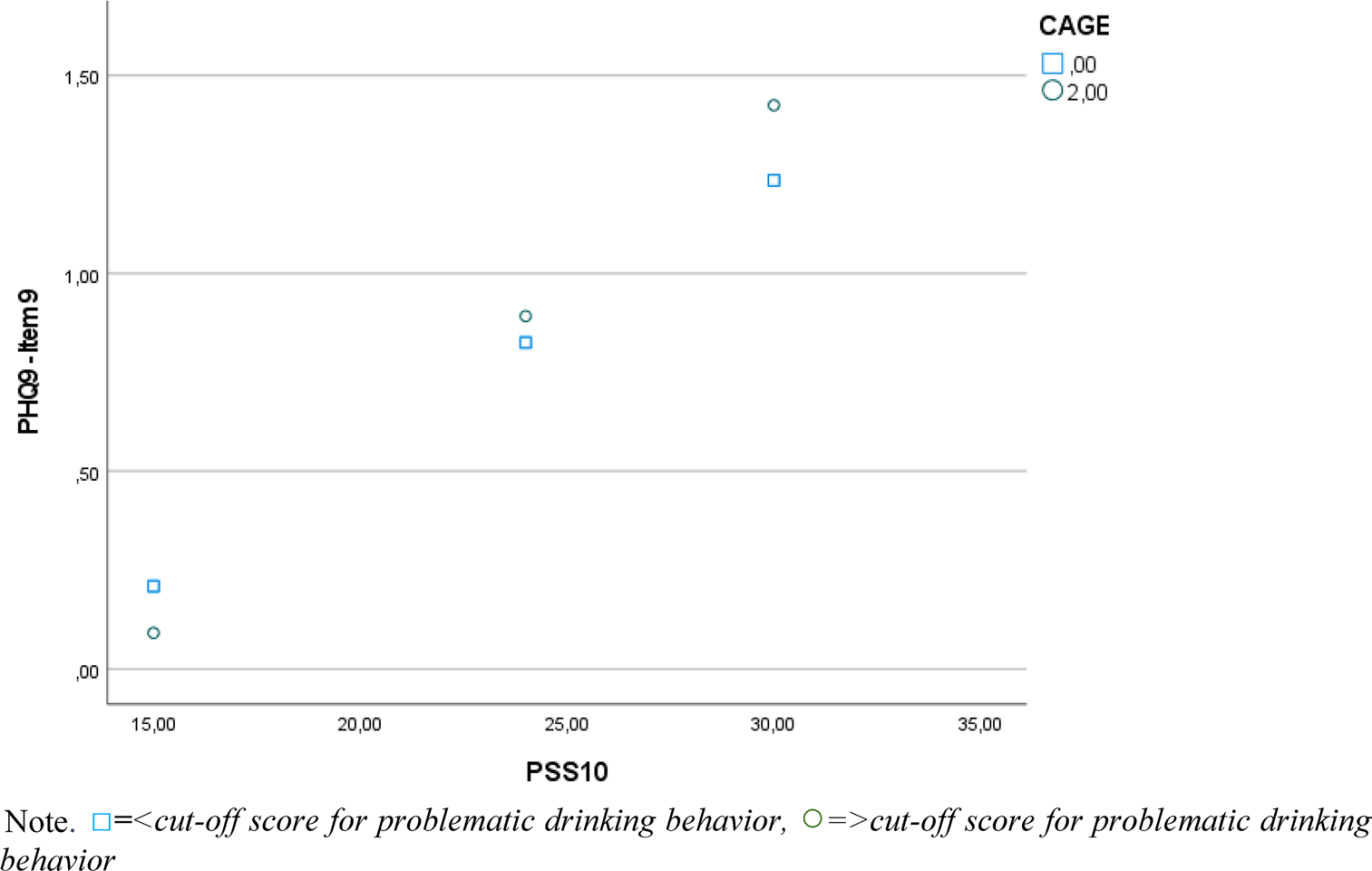
Moderation effect of problematic drinking behavior on the relationship between perceived stress and suicidal ideation.

A moderation analysis was run to determine whether the interaction between perceived stress and problematic drinking behavior significantly predicts suicidal ideation. The overall model was significant, F(5, 1499) = 144.122, *p* < 0.001, R = 0.570, predicting 32.47% of the variance. All predictors were significant before Bonferroni-adjustment (perceived stress = 0.072, *p* < 0.001, 95% CI [0.065, 0.078], problematic alcohol consumption = −0.188, *p* = 0.028, 95% CI [−0.355, −0.021]), but the *p*-value of problematic alcohol consumption surpassed the Bonferroni-adjusted significance level of 0.01666 Problematic alcohol consumption moderated the effect between perceived stress and suicidal ideation significantly, ΔR^2^ = 0.34%, F(1, 1499) = 7.50, *p* = 0.006, 95% CI [0.002, 0.016]. The R-Square change value indicates a small effect size. The interaction plot (Fig. 3) reveals that as stress levels increased, adolescents who showed signs of problematic drinking behavior (CAGE ≥ 2) reported higher suicidal ideation than those not meeting the criteria for problematic drinking behavior. There were no statistical significance transition points within the observed range of the moderator found using the Johnson-Neyman method, as the effect was significant for all observed moderator values.

## Discussion

Although neither physical activity, phone usage, nor problematic drinking behavior significantly predicted suicidal ideation on their own, all three health behaviors significantly moderated the relationship between perceived stress and suicidal ideation. Yet, the effects of the health behaviors were very small, indicating that the practical relevance of these results may be limited. Nevertheless, the results suggest that maintaining a healthy level of physical activity, spending little time on the phone, and reducing alcohol consumption may at least to a certain degree weaken the link between perceived stress and suicidal ideation. Based on the cross-sectional nature of this study, we cannot make any causal inferences on whether physical activity, phone usage, and problematic alcohol consumption influence stress or whether stress influences physical activity, phone usage, and problematic alcohol consumption. Nevertheless, the results suggest that the interplay between these health behaviors and stress has an, albeit small, effect on suicidal ideation. From a public health perspective, the pandemic related restrictions highlighted the urgent need for low-threshold interventions and adaptive health behaviors may at least have supportive effects on adolescents’ mental health during highly stressful times. Considering these results in the framework of the Narrative-Crisis Model of suicide^27^, physical activity may represent a protective factor for the development of the Suicidal Crisis Syndrome in vulnerable individuals experiencing stressful life events, while both excessive phone use and problematic alcohol consumption may represent risk factors in this regard. This is of particular interest, as the onset of the COVID-19 pandemic resulted in adverse changes in these health behaviors for a lot of young people^17,45^ Thus, focusing interventions and public health information on how to maintain a healthy lifestyle despite ongoing crises such as the COVID-19 pandemic, may pose an important factor for positive mental health change.

In line with the results of this study, under-exercising has been associated with higher stress levels and worse mental health, such as anxiety or depressive symptoms^15^, and greater amounts and higher intensities of physical activity generally seem to be more beneficial for physical and mental health^46^. However, not only the amount of time spent in physical activity is important, but the intensity as well. The World Health Organization guidelines for physical activity in children and adolescents recommend at least 60 min of moderate to vigorous physical activity daily for optimal health benefits^47^. A meta-analysis has identified beneficial effects of physical activity on mental health and suicidal ideation for adolescents, adults, and older adults alike^29^. Over-exercising, however, can be associated with health problems. It is well-known that over-exercising can be one symptom of eating disorders and poses a risk factor for developing an eating disorder^48^. Moreover, vigorous physical activity has been associated with higher accumulated concentrations of the stress-related hormone cortisol found in hair, compared to moderate physical activity^49^. In adolescents, over-exercising was linked to higher hair cortisol levels, compared to exercising the recommended amount^50^. While physical activity can be seen as a physical stressor, as it acutely increases circulating cortisol^51^, this does not necessarily correspond with an increase in subjectively perceived stress. On the contrary, higher physical activity has been linked to less perceived stress in adolescents^52^. Thus, a balanced approach to physical activity certainly is important for optimal physical and mental health. Adhering to the WHO physical activity guidelines, designed to facilitate a healthy lifestyle, is recommended and increasing adolescents’ motivation and competency for regular physical activity may be a fruitful approach to support their mental health, especially during times of crises, such as the COVID-19 pandemic.

Mobile phone use may be a form of coping with stress for adolescents and has become particularly relevant during the COVID-19 pandemic, when social distancing was necessary and many options for leisure-time activities were restricted. While multiple studies have reported an association between excessive phone use and negative mental health outcomes, it is important to note that in the daily lives of adolescents, and even more so during ongoing crises such as the COVID-19 pandemic, smartphones are an essential means of communicating and sharing information with each other^53^ and therefore may also hold potential for improving adolescents’ psychosocial health, when used responsively. As loneliness and social isolation have also been associated with increased suicidal ideation^25^, this is an important factor to consider when developing mental health interventions for this age group. Additional important factors in this regard, besides time spent on the phone, may be the consumed content as well as the way in which this content is consumed by adolescents. Making mental health information available online in an accessible format and developing digitally available interventions may hold a high potential for reaching this specific age group^54^. However, fostering public health awareness of responsible smartphone use is crucial to prevent harmful consequences of excessive smartphone use in adolescents.

Alcohol consumption can be classified as a maladaptive coping strategy^55^, as it can potentially have dose-dependent short-term stress reducing effects, especially in highly stressful situations^20^, but can lead to physiological stress-system dysregulations in the long-term^21^. Moreover, not only has problematic alcohol use been associated to suicidality^34^ and mental health problems such as depression and anxiety^56^. Considering the role of alcohol consumption in the relationship between stress and suicidal ideation, the duration of alcohol consumption seems critical. Responsible, short-term alcohol consumption should not increase suicidal ideation, but long-term use of alcohol to cope with stressful situations can exacerbate stress and mental health problems and in turn increase suicidal ideation. Chronic alcohol abuse also significantly increases overall mortality^57^, by increasing the risk of various cancers, liver disease, hypertension, and, especially relevant for younger people, also injuries and violence^58^. A systematic review on alcohol consumption in 15-to-19-year-olds suggests that reducing problematic alcohol consumption during adolescence might prevent negative long-term consequences of alcohol consumption for physical and mental health in later years, as well as harmful short-term consequences, such as injuries through accidents^59^.

For researchers and practitioners alike, it might be of interest to examine physical activity, phone usage, and problematic alcohol consumption as points of action in public health interventions targeting suicidality in adolescents suffering from the consequences of the COVID-19 pandemic, or other ongoing crises.

### Limitations

Some limitations to the study need to be considered when interpreting the results. Most importantly, the cross-sectional nature of the survey does not allow for any causal conclusions. Therefore, a longitudinal investigation of the effects proposed in this study is vital. Secondly, the online nature of the survey, although enabling the collection of a large sample, carries the potential to introduce self-selection bias. Adolescents without access to the internet could not be reached this way. The gender distribution was not representative of the Austrian population, with more girls taking part in the study than boys. Furthermore, only self-report measures of physical activity, phone usage, and stress were included, whereas accelerometric data could provide more objective measures of these variables. Suicidal ideation was only assessed with a single item question in PHQ 9 and not with a standardized questionnaire. Lastly, CAGE results from non-clinical samples should be interpreted cautiously^40^.

## Conclusions

Overall, these results suggest that some health behaviors may hold potential to attenuate the effect of stress on suicidal ideation, at least to a certain degree, while adverse health behaviors may even exacerbate it. However, as this cross-sectional design does not allow for any causal inferences, further exploration of these proposed relationships in longitudinal studies is necessary. Although revealing only small effects, these results seem highly relevant, not only from a public health perspective. Health behavior can be changed and should be actively promoted. Thus, evaluated low-threshold interventions to support adolescents’ mental health during ongoing crises are urgently needed. Increasing physical activity, decreasing phone time or reducing alcohol consumption might be viable as such and should be investigated further in longitudinal studies. While systemic effort is necessary to prevent adverse developments in adolescent mental health in the future, taking action in one’s everyday life by getting enough exercise, reducing phone use, and reducing alcohol intake may alleviate some of the impact of perceived stress on suicidal ideation and act as a protective factor for adolescents’ mental health during ongoing crises.

## Data Availability

The datasets used and analyzed during this study are available from the corresponding author on reasonable request.
